# Carbohydrate Metabolism in Bacteria: Alternative Specificities in ADP-Glucose Pyrophosphorylases Open Novel Metabolic Scenarios and Biotechnological Tools

**DOI:** 10.3389/fmicb.2022.867384

**Published:** 2022-04-27

**Authors:** Jaina Bhayani, Maria Josefina Iglesias, Romina I. Minen, Antonela E. Cereijo, Miguel A. Ballicora, Alberto A. Iglesias, Matias D. Asencion Diez

**Affiliations:** ^1^Department of Chemistry and Biochemistry, Loyola University Chicago, Chicago, IL, United States; ^2^Facultad de Bioquímica y Ciencias Biológicas, Instituto de Agrobiotecnología del Litoral, Universidad Nacional del Litoral, Consejo Nacional de Investigaciones Científicas y Técnicas, Santa Fe, Argentina

**Keywords:** glucose-1-phosphate, glucosamine-1-phosphate, glucosamine-6-phosphate, allosterism, promiscuity

## Abstract

We explored the ability of ADP-glucose pyrophosphorylase (ADP-Glc PPase) from different bacteria to use glucosamine (GlcN) metabolites as a substrate or allosteric effectors. The enzyme from the actinobacteria *Kocuria rhizophila* exhibited marked and distinctive sensitivity to allosteric activation by GlcN-6P when producing ADP-Glc from glucose-1-phosphate (Glc-1P) and ATP. This behavior is also seen in the enzyme from *Rhodococcus* spp., the only one known so far to portray this activation. GlcN-6P had a more modest effect on the enzyme from other Actinobacteria (*Streptomyces coelicolor*), Firmicutes (*Ruminococcus albus*), and Proteobacteria (*Agrobacterium tumefaciens*) groups. In addition, we studied the catalytic capacity of ADP-Glc PPases from the different sources using GlcN-1P as a substrate when assayed in the presence of their respective allosteric activators. In all cases, the catalytic efficiency of Glc-1P was 1–2 orders of magnitude higher than GlcN-1P, except for the unregulated heterotetrameric protein (GlgC/GgD) from *Geobacillus stearothermophilus*. The Glc-1P substrate preference is explained using a model of ADP-Glc PPase from *A. tumefaciens* based on the crystallographic structure of the enzyme from potato tuber. The substrate-binding domain localizes near the N-terminal of an α-helix, which has a partial positive charge, thus favoring the interaction with a hydroxyl rather than a charged primary amine group. Results support the scenario where the ability of ADP-Glc PPases to use GlcN-1P as an alternative occurred during evolution despite the enzyme being selected to use Glc-1P and ATP for α-glucans synthesis. As an associated consequence in such a process, certain bacteria could have improved their ability to metabolize GlcN. The work also provides insights in designing molecular tools for producing oligo and polysaccharides with amino moieties.

## Introduction

α-1,4-Glucans are biomolecules produced in several types of bacteria, including Gram-positive, Gram-negative, cyanobacteria, and archaebacteria (Preiss, 1984, 2009, 2014; Preiss and Romeo, 1990). Glycogen, an important α-glucan polysaccharide in bacteria, is synthesized by the classical *glgC*-*glgA* pathway, where ATP activates glucose-1P (Glc-1P) into ADP-Glc, the glycosyl donor for glucan elongation. These reactions are, respectively, catalyzed by ADP-Glc pyrophosphorylase (ADP-Glc PPase, EC 2.7.7.27) and glycogen synthase (EC 2.4.1.21). The pathway completes the formation of glycogen *via* the branching enzyme (EC 2.4.1.18), which introduces α-1,6-ramifications (Preiss, 2009; Cifuente et al., 2019). In this sequence of reactions, the limiting step is at the level of ADP-Glc PPase, which is regulated by metabolites from the major carbon utilization route in the organism (specified below) (Ballicora et al., 2003, 2004; Figueroa et al., 2021, 2022).

ADP-Glc PPases from numerous bacterial sources have been characterized concerning kinetic, regulatory, and structural properties (Ballicora et al., 2003, 2004; Figueroa et al., 2022). In Proteobacteria and Actinobacteria, the enzyme is a tetramer composed of a single subunit (codified by the *glgC* gene) and is allosterically regulated by critical intermediates of the central metabolism in the respective microorganism. For example, fructose-1,6-bisphosphate or fructose-6-phosphate (Fru-6P) and pyruvate (Pyr) are major activators of the enzyme from *Escherichia coli* (performing glycolysis by the Embden–Meyerhof pathway) and *Agrobacterium tumefaciens* (using the Entner–Doudoroff route), respectively (Ballicora et al., 2003, 2004). Also, the ADP-Glc PPase from Actinobacteria has multiple allosteric activators, notably glucose-6-phosphate (Glc-6P) (Asencion Diez et al., 2012, 2015; Cereijo et al., 2016, 2020), agreeing with the varied metabolic pathways having the hexose-P as a common starting point (Verma et al., 2013; Martín and Liras, 2020). Conversely, in Firmicutes (Gram-positive bacteria having low G + C content), two genes (*glgC* and *glgD*) codify for ADP-Glc PPase, giving rise to tetrameric GlgC or GlgC/GlgD active forms (Takata et al., 1997; Asención Diez et al., 2013a; Cereijo et al., 2018). GlgD is a protein lacking activity and is absent in Actinobacteria and Proteobacteria (Asención Diez et al., 2013a). The enzyme (either GlgC or GlgC/GlgD forms) from *Geobacillus stearothermophilus* (and the Bacillales group) is the only known ADP-Glc PPase exhibiting insensitivity to allosteric regulators to our knowledge (Takata et al., 1997). In other Firmicutes (Lactobacillales and Clostridiales), GlgC and GlgC/GlgD have distinct responses to specific allosteric regulators with the GlgD subunit providing a modulatory function (Asención Diez et al., 2013a; Cereijo et al., 2018). For example, the GlgC/GlgD from *Ruminococcus albus* exhibits higher catalytic efficiency than GlgC and phospho*enol*pyruvate (PEP) is the primary allosteric activator (Cereijo et al., 2018).

The context detailed above supports that ADP-Glc PPase was a target of evolution (Ballicora et al., 2003; Figueroa et al., 2022). This protein acquired sensitivity to varied allosteric effectors (based on the different metabolic pathways in diverse prokaryotes) to orchestrate the regulation of its activity. ADP-Glc PPase distinguishes itself from all other pyrophosphorylases because of its sensitivity to allosteric regulators. This modulatory characteristic is structurally justified by having a C-terminal (absent in other pyrophosphorylases) critical for regulatory function. It has been demonstrated that the tight interaction between the C- and the N-(catalytic) domains (Bejar et al., 2004) exerts allosteric control *via* a mechanism triggered by conformational changes of specific motifs in the enzyme (Figueroa et al., 2011, 2013; Asención Diez et al., 2013b, 2020). A detailed study (Ebrecht et al., 2017) evidenced that the ADP-Glc PPase from *E. coli* exhibits relative promiscuity for substrates (a common trait amongst catalytic proteins) (Jensen, 2003; Dönertaş et al., 2016; Copley and Copley, 2020) and that allosteric activation primarily drives the enzyme to select the production of ADP-Glc from Glc-1P and ATP. In other words, the evolutionary mechanism developed to regulate ADP-Glc PPases operated by adapting the protein to select specific substrates (Ebrecht et al., 2017).

Recent works have pointed out the potential relevance of glucosamine (GlcN, 2-Amino-2-deoxy-glucose) metabolism in actinobacteria of the genus *Rhodococcus* (Cereijo et al., 2016, 2020, 2021). ADP-Glc PPase from rhodococci can utilize GlcN-1P as a substrate and: (*i*) allosteric effectors enhance this activity and (*ii*) GlcN-6P is a primary allosteric activator (Cereijo et al., 2020). These findings were particularly relevant relating evolution and the mechanism for allosteric properties exhibited by this enzyme. These data challenged us to perform studies to explore the sensitivity of ADP-Glc PPases from other bacteria to the GlcN-P metabolites. Herein, we show that GlcN-6P is a common allosteric effector, with significant effect on the protein from Actinobacteria. In addition, GlcN-1P is a suitable substrate, giving rise to different catalytic efficiencies for the enzyme from Actinobacteria, Firmicutes, and Proteobacteria. Using the elucidated crystallographic structure of the enzyme from potato tuber as a starting model, we placed Glc-1P and GlcN-1P in the active site of the *A. tumefaciens* enzyme to discuss the metabolic evolution of GlcN in ADP-Glc PPase from prokaryotes. Finally, based on distinct enzymatic behavior, we discuss some putative applications where ADP-Glc PPases could be incorporated in the design and production of oligo- and polysaccharides with potential biotechnological interests.

## Materials and Methods

### Chemicals, Bacterial Strains, and Plasmids

Chemicals used for enzymatic assays were from Sigma-Aldrich (St. Louis, MO, United States) or the highest quality available. *E. coli* Top 10 (Invitrogen) was used for plasmid maintenance, and proteins were expressed in *E. coli* BL21 (DE3) (Invitrogen) using the pET28b vector (Novagen). DNA manipulations, molecular biology techniques, and *E. coli* cultivation and transformation were performed according to standard protocols (Sambrook and Russell, 2001).

### Site-Directed Mutagenesis

Mutagenesis was performed by using a Q5 Site-Directed Mutagenesis Kit from New England BioLabs. Oligonucleotides were synthesized by Integrated DNA Technologies (IDT, San Diego). The primers listed were used to make E187A in ADP-Glc PPase from *A. tumefaciens*: E187A Forward: CGACTTCATCGCCAAGCCGGC and E187A Reverse: ATGATCTCGTCTTTTTCGTTCACATGC.

### Enzyme Production and Purification

Proteins were obtained as previously described (Takata et al., 1997; Asencion Diez et al., 2012, 2020; Cereijo et al., 2018, 2020). Briefly, the general procedure involved a recombinant *E. coli* BL21 (DE3) (Invitrogen) transformed with a recombinant plasmid [in most cases, pET28b vector (Novagen)] harboring the desired *glgC* gene. Transformed cells were cultured at 37°C and 200 rpm in LB medium (10 g/l tryptone; 5 g/l yeast extract; and 10 g/l NaCl) containing the corresponding antibiotics at its proper concentration [kanamycin (50 μg/ml); ampicillin (100 μg/ml)] until optical density at 600 nm reached between ~0.6 and 0.8. Then, protein expression was induced by adding 0.1–0.4 mM isopropyl-β-D-1-thiogalactopyranoside (depending the protein), which continued for 16 h at 18°C–24°C. Then, recombinant cells were harvested with a 10-min centrifugation at room temperature and 5,000 × *g*, and the obtained pellets were stored at −20°C until used for purification purposes.

Immobilized metal affinity chromatography (IMAC) at 4°C was the procedure to purify the His-tagged proteins. Protein purification started with cells resuspension in *Buffer H* [50 mM Tris–HCl pH 8.0, 300 mM NaCl, 10 mM imidazole, and 5% (v/v) glycerol], disruption by sonication (4 s on; 2 s off, for a total time of 10 min in an ice-bath), and then centrifuging twice at 30,000 × *g* for 10 min. The resulting supernatant (crude extract) was loaded on a His-Trap column (1 ml size, GE Healthcare) that had been equilibrated with *Buffer H*. Elution of the recombinant proteins was produced using a linear imidazole gradient (10–300 mM) in the same buffer. Fractions exhibiting the highest ADP-Glc PPase activity were pooled and concentrated. After concentration, the ADP-Glc PPases from *Streptomyces coelicolor*, *Rhodococcus fascians*, and *R. albus* were dialyzed against *Buffer S* [50 mM HEPES-NaOH, 10% (w/v) sucrose, 0.2 mM DTT, and 1 mM EDTA]. The resulting purified enzymes were stored at −80°C until use. Under these conditions, enzymes remained active for 6 months (at least).

### Protein Methods

Protein concentration was assayed by the Bradford method (Bradford, 1976), with the use of bovine serum albumin (BSA) as a standard. The purity of the recombinant proteins was assessed by sodium dodecyl sulfate–polyacrylamide gel electrophoresis (SDS-PAGE), as described elsewhere (Laemmli, 1970). After running, the gels (containing between 5 and 50 μg of protein per well) were revealed with Coomassie Brilliant Blue.

### Enzyme Activity Assays

The ADP-Glc PPase activity was determined at 37°C in the direction of ADP-Glc synthesis following the formation of P_i_ after PP_i_ hydrolysis by inorganic pyrophosphatase, using the colorimetric method described before (Fusari et al., 2006). Reaction mixtures (a total volume of 50 μl) contained (unless otherwise specified) 50 mM MOPS-NaOH pH 8.0, 1–2 mM ATP, 10 mM MgCl_2_, 0.5 U/ml yeast inorganic pyrophosphatase, 0.2 mg/ml BSA, and a proper enzyme dilution. The addition of 1–2 mM Glc-1P initiated the assays, which were run for 10 min, and terminated by the addition of 400 μl of the Malachite Green reagent. The complex formed with the released P_i_ was measured at 620 nm in a 96-well microplate reader (Multiskan GO, Thermo).

One unit of activity (U) is defined as the amount of enzyme catalyzing the formation of 1 μmol of product per min under the above-specified conditions.

Saturation curves were constructed by assaying enzyme activity at different concentrations of the variable substrate or effector, while the others remained at saturating levels. Plots of enzyme activity (U/mg) vs. substrate (or effector) concentration (mM) were used to calculate the kinetic constants by fitting the experimental data to a modified Hill equation (Ballicora et al., 2007). Data were fitted using the Levenberg–Marquardt nonlinear least-squares algorithm included in the computer program Origin 8.0 (OriginLab). Accordingly, we calculated the maximal velocity (*V*_max_), the Hill coefficient (*n*_H_), and the concentrations of activator (*A*_0.5_) or substrate (*K*_m_), giving 50% of the maximal activation or velocity, respectively. Kinetic constants were reproducible within a range of ±10%, being the mean of three independent sets of data.

### Computational Methods

The *A. tumefaciens* ADP-Glc PPase structure with ADP-Glc was built using the following templates: the crystal structure of the small subunit of the potato (*Solanum tuberosum*) tuber in complex with ADP-Glc (PDB ID: 1YP4) and *A. tumefaciens* ADP-Glc PPase (PDB ID: 5W5R; Jin et al., 2005; Hill et al., 2019). The model was generated using MODELLER (Šali and Blundell, 1993; Eswar et al., 2007). Protein visualization was performed with the program Chimera (Pettersen et al., 2004).

## Results

### GlcN-6P as a Bacterial ADP-Glc PPase Activator

**Figure 1:**
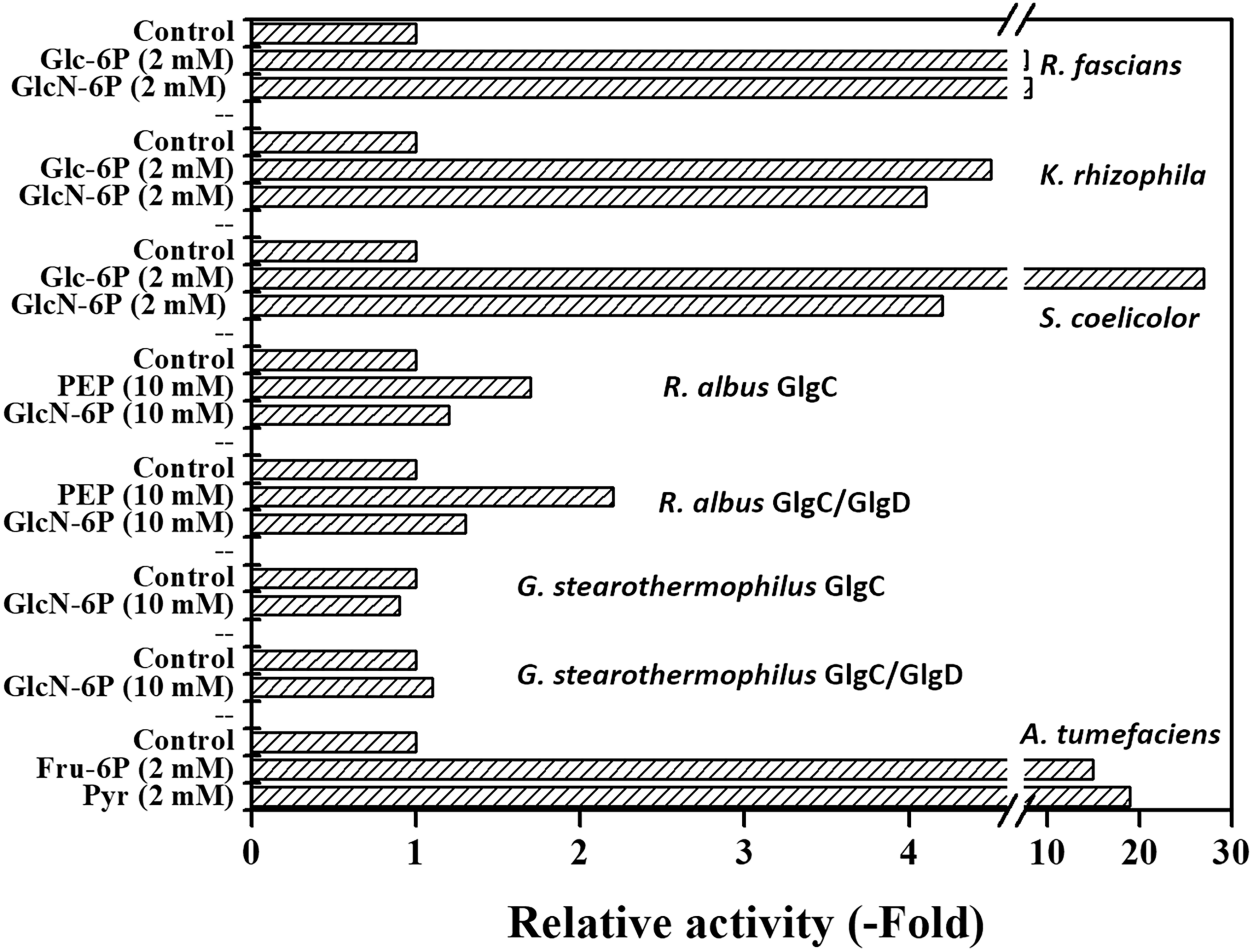
GlcN-6P as effector of bacterial ADP-glucose pyrophosphorylases (ADP-Glc PPases). Activities were measured according to the section “Materials and Methods,” using indicated effector concentrations.

Recently, it was found that GlcN-6P is a primary activator of the rhodococcal ADP-Glc PPase, improving the catalytic properties of both the canonical and the alternative substrates, Glc-1P and GlcN-1P, respectively (Cereijo et al., 2020). Given that GlcN-6P was reported as a key metabolite in some Gram-positive microorganisms (Jolly et al., 2000; Urem et al., 2016; van der Heul et al., 2018; Manso et al., 2019), we tested whether other ADP-Glc PPases from Actinobacteria, *S. coelicolor* and *Kocuria rhizophila*, are sensitive to the glucosamine derivative. GlcN-6P increased 4-fold the Glc-1P activity of the *S. coelicolor* enzyme (Figure 1; *A*_0.5_ 3.55 ± 0.52 mM), which was a small effect compared to the 27-fold activation exerted by the major effector Glc-6P (Asencion Diez et al., 2012). On the other hand, Figure 1 shows that the *K. rhizophila* ADP-Glc PPase responded to the GlcN-6P stimulus to the same extent as to Glc-6P (4–5-fold increase), thus behaving similarly to the rhodococcal enzyme (Cereijo et al., 2020). In these experiments, the *R. fascians* enzyme was activated about 8-fold with either GlcN-6P or Glc-6P. Therefore, GlcN-6P could be ascribed as a common activator in actinobacterial ADP-Glc PPases, with varying intensities, depending on the source. Interestingly, the *S. coelicolor* ADP-Glc PPase is the only enzyme from this group showing a distinct level of activation for various molecules (Asencion Diez et al., 2012), since ADP-Glc PPases from *Mycobacterium tuberculosis* (Asencion Diez et al., 2015), *Rhodococci* (Cereijo et al., 2020), and *K. rhizophila* exhibit similar degrees of activation for each effector.

We then analyzed the GlcN-6P activation of the different quaternary structures (GlgC or GlgC/GlgD) of the ADP-Glc PPase from two Firmicutes species: *R. albus* and *G. stearothermophilus*. Both GlgC and GlgC/GlgD enzymes from *G. stearothermophilus* were insensitive to GlcN-6P (Figure 1), even when assayed at 10 mM, in agreement with the observed recalcitrant insensitivity of these proteins to regulation, constituting the exception among all ADP-Glc PPases (Takata et al., 1997; Ballicora et al., 2003; Asención Diez et al., 2013a; Cereijo et al., 2018). In contrast, both *R. albus* GlgC and GlgC/GlgD responded to GlcN-6P activation, although to a lesser extent (about one half) than their main effector PEP (Cereijo et al., 2018), as shown in Figure 1. We also assayed the effect of GlcN-6P on the *A. tumefaciens* ADP-Glc PPase and curiously found that it decreased activity about 30%, while allosteric controls with Fru-6P and Pyr increased activity 15–20-fold (Uttaro et al., 1998; Asencion Diez et al., 2020). Taken together, our results show that all regulated ADP-Glc PPase analyzed here responded to GlcN-6P to some extent.

### GlcN-1P as a Substrate for ADP-Glc PPase

**Figure 2:**
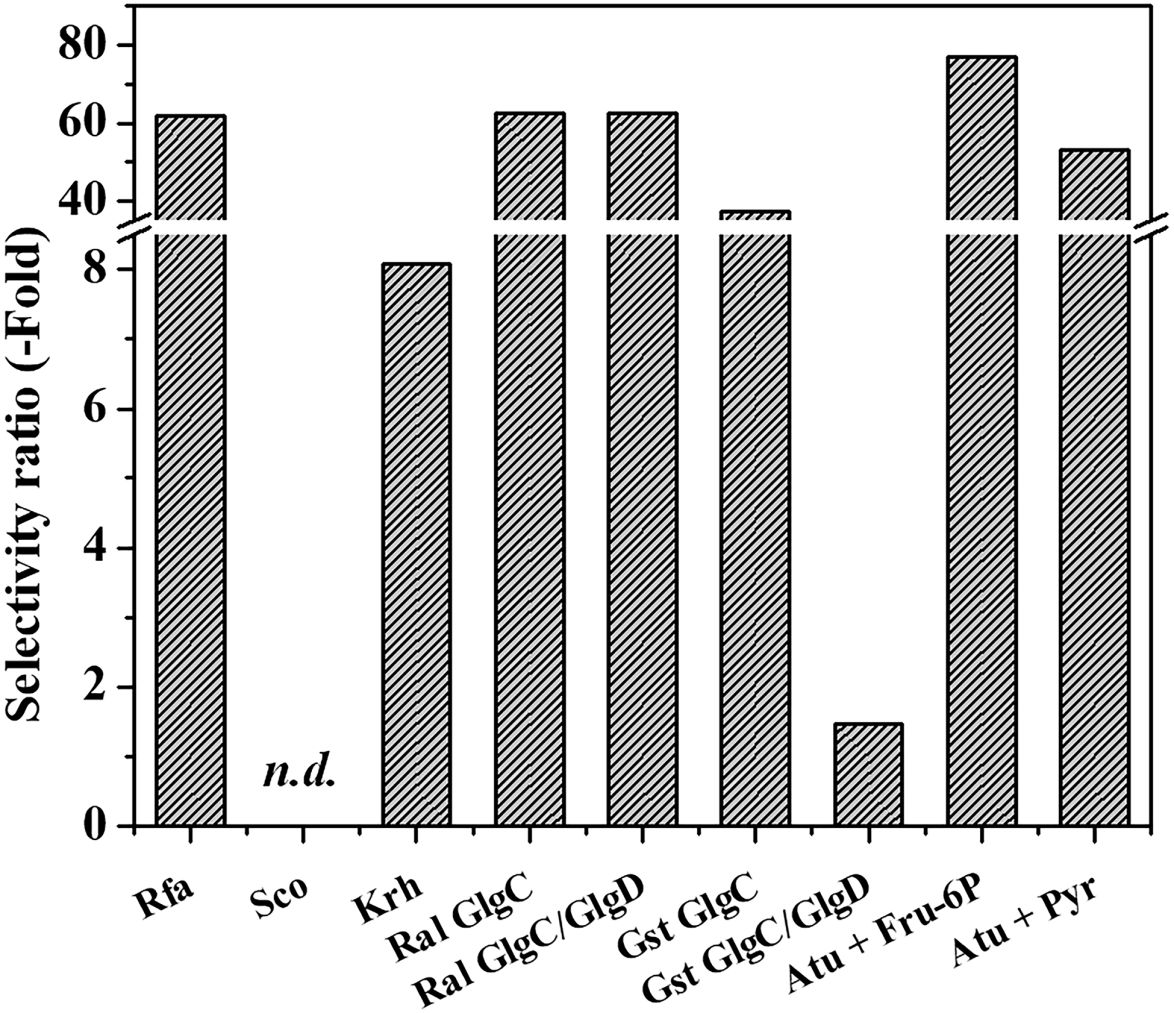
Selectivity ratios for glucose-1-phosphate (Glc-1P) and GlcN-1P (ratio between catalytic efficiency for the use of Glc-1P over that with GlcN-1P).

Recently, two NDP-sugar pyrophosphorylases were characterized with the ability to use GlcN-1P as substrate: one being specific for the amino sugar-1P and UTP (GalU2; Cereijo et al., 2021) and the other using GlcN-1P as an alternative substrate with ATP (Cereijo et al., 2020). These enzymes belong to the *Rhodococcus* genus and are the only known studies regarding GlcN-1P consumption (for further details, see Cereijo et al., 2021). Therefore, we analyzed GlcN-1P as a substrate for ADP-Glc PPases from different bacterial sources in the presence of their main activators as well as GlcN-6P. When possible, parameters were also obtained without effectors, particularly for the allosterically insensitive *G. stearothermophilus* GlgC and GlgC/GlgD enzymes. Selectivity ratios, defined as the catalytic efficiencies of Glc-1P divided by GlcN-1P in the different bacterial ADP-Glc PPases, are shown in Figure 2. General kinetic parameters for the characterized enzymes are presented in Supplementary Table 1.

First, we studied GlcN-1P utilization by ADP-Glc PPase from the actinobacteria *S. coelicolor* and *K. rhizophila*. Both were active with the alternative substrate, although with values in the mU/mg order of magnitude (9 and 30 mU/mg for the enzymes from *S. coelicolor* and *K. rhizophila*, respectively). The *S. coelicolor* ADP-Glc PPase poorly used GlcN-1P and only showed a low activation degree in the presence of Glc-6P and GlcN-6P. GlcN-6P barely doubled the specific activity, and while Glc-6P increased activity 4-fold, it remained in the mU/mg order (Supplementary Figure 1). In contrast, the *K. rhizophila* enzyme behaved similarly to the rhodococcal ADP-Glc PPases (Cereijo et al., 2020), responding to a 2-mM GlcN-6P stimulus for both the sugar phosphate and amino-sugar phosphate as substrates. With Glc-1P, the activator increased the *V*_max_ 4-fold with an *A*_0.5_ value of 0.08 mM. Remarkably, GlcN-6P (with an *A*_0.5_ of 0.84 mM) increased GlcN-1P activity by two orders of magnitude, reaching up to ~20 U/mg. In terms of catalytic efficiency, the *K. rhizophila* ADP-Glc PPase used GlcN-1P with an efficiency that is almost one order of magnitude lower compared to Glc-1P (Figure 2). However, the efficiency of the *Kocuria* enzyme for GlcN-1P is 16-fold higher than the one of rhodococcal ADP-Glc PPases, making it a better biocatalyst tool in this respect (Supplementary Table 1).

We also explored the ability of the GlgC or GlgC/GlgD structures of the *G. stearothermophilus* ADP-Glc PPase to use GlcN-1P as an alternative substrate. With this substrate (at 1 mM), we observed 10% activity relative to Glc-1P (not shown), similar to the rhodococcal ADP-Glc PPase (Cereijo et al., 2016, 2020). The GlgC homotetramer had a catalytic efficiency of 0.36 mM^−1^ s^−1^ toward GlcN-1P, a value in the same order of magnitude as the rhodococcal enzymes (between 0.3 and 0.5 mM^−1^ s^−1^) (Cereijo et al., 2020). It is important to note that the efficiency for these substrates in the *G. stearothermophilus* protein was in the absence of activator and the catalytic efficiency for GlcN-1P is 37-fold lower than Glc-1P (Figure 2), sustained by lower values for both maximum activity and apparent affinity. Interestingly, the selectivity ratio decreased to 1.5 for the heterotetramer GlgC/GlgD (Figure 2) showing that the presence of both subunits diminishes substrate selectivity. The presence of GlgD subunit increased both the catalytic capacity (around 20-fold) and the apparent affinity (*K*_m_ ~ 0.4 mM) for GlcN-1P. Furthermore, in the presence of 1 mM GlcN-1P, ATP reached similar *k*_cat_ and *K*_m_ (0.21 mM) levels (Supplementary Figure 2) with a catalytic efficiency for ATP utilization with GlcN-1P at 40.28 mM^−1^ s^−1^, a value 7-fold higher than with Glc-1P, according to data previously reported (Takata et al., 1997).

Therefore, the *G. stearothermophilus* GlgC/GlgD heterotetrameric structure of ADP-Glc PPase has the highest reported catalytic efficiency to use ATP and GlcN-1P allowing us to hypothesize that it may be metabolically meaningful (Bar-Even et al., 2011; Davidia et al., 2016). Also important, at a catalytic level, is the effect of the inactive GlgD subunit on the global behavior of ADP-Glc PPase, which is not observed for the substrate Glc-1P (Takata et al., 1997). The homotetrameric GlgC enzyme from the *R. albus* used GlcN-1P with a lower efficiency than its canonical substrate Glc-1P (selectivity ratio of ~60; Figure 2). The heterotetrameric GlgC/GlgD from *R. albus* also utilized GlcN-1P as an alternative substrate, even in the absence of activator. This is like the *G. stearothermophilus* enzymes presented above, but it marks a difference with the actinobacterial ADP-Glc PPases, which need to be activated. The heteromeric GlgC/GlgD from *R. albus* also showed 60-fold lower catalytic efficiency toward GlcN-1P than for Glc-1P (Figure 2). Although the enzyme shows a *k*_cat_ with the amino sugar-1P that is 70% of that with the sugar-1P, the enzyme possesses a low apparent affinity (~2 mM) for GlcN-1P. The catalytic efficiency for GlcN-1P in the heterotetrameric *G. stearothermophilus* enzyme falls within the range of a plausible and meaningful metabolic reaction, according to the hypothesis presented elsewhere (Bar-Even et al., 2011; Davidia et al., 2016).

Complementing the study of GlcN-1P utilization by bacterial ADP-Glc PPase, we analyzed this activity using the model enzyme from *A. tumefaciens*, which has been thoroughly analyzed at the kinetic, regulatory, and structural levels (Uttaro and Ugalde, 1994; Uttaro et al., 1998; Gómez-Casati et al., 2001; Hill et al., 2019; Asencion Diez et al., 2020). The enzyme was active with GlcN-1P as a substrate, but its efficiency was about two orders of magnitude lower than Glc-1P. Both Fru-6P and Pyr activated GlcN-1P utilization (remarkably, the enzyme resulted highly sensitive to activation, with an *A*_0.5_ between 27 and 33 μM for both effectors) exhibiting selectivity ratios with respect to Glc-1P in the range of 50–80 (Figure 2). We observed that the apparent affinity for ATP remains constant whether the other substrate is Glc-1P or GlcN-1P. Considering the availability of the crystallographic structure of the *A. tumefaciens* ADP-Glc PPase and that the enzyme used GlcN-1P with a lower preference than the canonical substrate, it was relevant to explore further this kinetic characteristic in the framework of the protein structure.

### Structural Approach to GlcN-1P Binding/Catalysis

**Figure 3:**
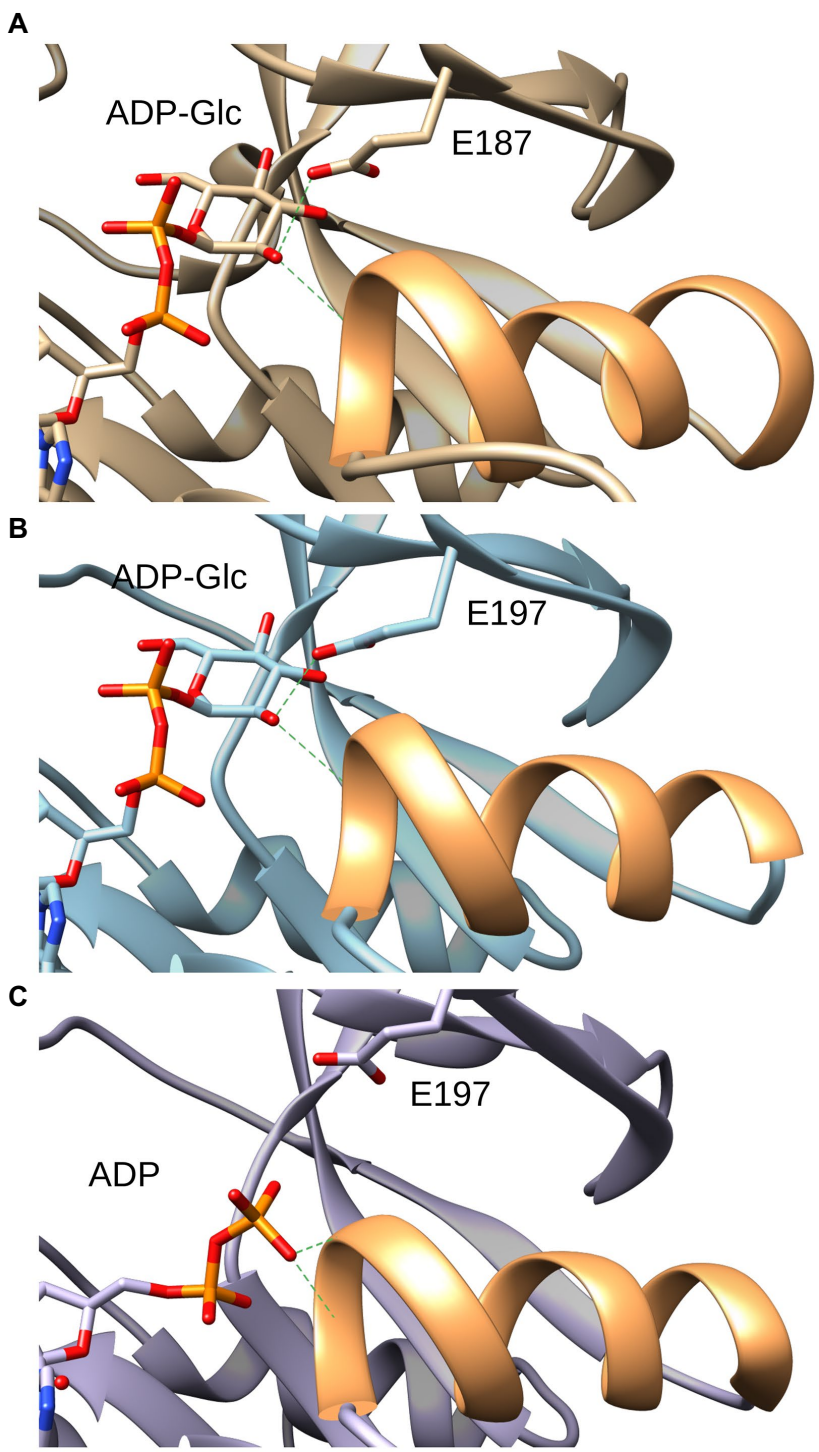
Interaction of the glucose moiety of the product ADP-Glc with the ADP-Glc PPase from *Agrobacterium tumefaciens* and potato tuber. **(A)** Structure of the *A. tumefaciens* ADP-Glc PPase in which ADP-Glc has been modeled as described in the section “Materials and Methods.” In orange is the ribbon corresponding to the α-helix from residues F233 to H244. Green dashes indicate the distance from the OH (position 2) to the side chain oxygen of the E187 residue (3.1 Å) and to the backbone N of the residue G234 (4.0 Å), respectively. **(B)**: Structure of the small subunit ADP-Glc PPase from potato tuber (PDB ID: 1YP4, subunit B) with ADP-Glc in the active site. In orange is the ribbon corresponding to the α-helix from residues F254 to S264. Green dashes indicate the distance to the O of side chain of the E197 residue (2.8 Å) and to the N of the backbone of the residue G255 (4.0 Å), respectively. **(C)** Structure of the small subunit ADP-Glc PPase from potato tuber (PDB ID: 1YP4, subunit A) with ADP in the active site. In orange is the ribbon corresponding to the α-helix from residues F254 to S264. Green dashes indicate the distance from the closest O of the β-phosphate of ADP to the backbone nitrogen of the S256 residue (3.3 Å) and to the backbone nitrogen of the residue G255 (3.9 Å), respectively.

The only structural difference between Glc-1P and GlcN-1P is that an amino group is in position 2 rather than a hydroxyl group. Therefore, to understand the selectivity between Glc-1P and GlcN-1P in ADP-Glc PPases, it is important to analyze the structural determinants for the binding of the hydroxyl group in position 2 when Glc-1P is the substrate. Using the elucidated crystallographic structure of ADP-Glc PPase from potato tuber, we modeled the sugar-phosphate binding site in *A. tumefaciens*. Previous studies in crystal structures of proteins have revealed that alcohol binding sites generally consist of a hydrogen bond acceptor in a turn or loop region that is often situated at the N-terminal end of an α-helix (Dwyer and Bradley, 2000). This is certainly the case in the small subunit of the ADP-Glc PPase from potato tuber (Figure 3). The hydroxyl group in position 2 of the glucosyl moiety of ADP-Glc, which is the product after Glc-1P reacted with ATP, is in a perfect place to donate hydrogen to the carboxylate of E197 (Jin et al., 2005). In addition, this binding site is near the N-terminal end of the α-helix comprising residues between G255 and L265. No other interactions are apparent. Structural analysis of the *A. tumefaciens* enzyme, where ADP-Glc has been modeled (Figure 3), yielded identical results. Therefore, it is expected that these two major structural elements, the H-bond acceptor E187 (E197 in potato tuber, E194 in *E. coli*) and the α-helix, will contribute to selecting a hydroxyl over an amino group, and as such, prefer Glc-1P over GlcN-1P.

A hypothesis was that the difference in strength between an H-bond could explain the difference in substrate specificity. This is because the oxygen from a hydroxyl group is predicted to be a stronger donor than nitrogen from a primary amine (Halder et al., 2019). In both cases, the acceptor is one of the oxygens from the carboxylate (E187). If this hypothesis is true, the elimination of the acceptor by mutagenesis should remove or significantly decrease the differences between Glc-1P and GlcN-1P. Previously, the homologous glutamic acid at position 194 was mutated to alanine in the *E. coli* ADP-Glc PPase and was found to be critical to the binding of Glc-1P (Bejar et al., 2006). We mutated E187 to alanine to determine whether we can eliminate the substrate preference of Glc-1P over GlcN-1P. In the E187A mutant with no effector, Glc-1P had an *K*_m_ of 0.4 mM, nearly 4.5-fold worse than the wild-type (WT) enzyme (Table 1). In the presence of its major activators, the *K*_m_ increased 13-fold for Fru-6P and 12-fold for Pyr for E187A compared to the WT, showing that the binding of the substrate is greatly hindered by removing the negatively charged glutamic acid residue with alanine. When comparing the catalytic efficiencies between Glc-1P and GlcN-1P without an activator, there is still a 79-fold preference for Glc-1P over GlcN-1P in E187A. Even with activators, the mutated enzyme still shows selectivity for Glc-1P over GlcN-1P, meaning that the glutamic acid does not solely control the preference of sugar-phosphate for this enzyme.

**Table 1:** Kinetic parameters for E187A ADP-Glc PPase from *A. tumefaciens* with Glc-1P and GlcN-1P as substrates in the absence and presence of activators.

		Glc-1P			GlcN-1P		Selectivity ratio
	$V_{\max}$	*K*_m_	VmaxKm	$V_{\max}$	*K*_m_	VmaxKm	VmaxKmGlc1PVmaxKmGlcN1P
	(U·mg^−1^)	(mM)	(U·mg^−1^·mM^−1^)	(U·mg^−1^)	(mM)	(U·mg^−1^·mM^−1^)	
No Effector	0.27 ± 0.01	0.427 ± 0.049	0.632	0.012 ± 0.001	1.50 ± 0.23	0.008	79
Fru-6P (1.5 mM)	4.00 ± 0.15	0.56 ± 0.05	7.14	0.193 ± 0.025	1.83 ± 0.37	0.105	68
Pyr (1.5 mM)	3.85 ± 0.13	0.65 ± 0.05	5.92	0.062 ± 0.002	0.93 ± 0.10	0.067	88
GlcN-6P (1.5 mM)	0.208 ± 0.004	1.42 ± 0.04	0.146	0.005 ± 0.0004	0.78 ± 0.15	0.006	24

## Discussion

Pyrophosphorylases (PPases) are critical enzymes for carbohydrate metabolism in different organisms, catalyzing the reversible production of nucleoside-diphospho-sugar and their derivatives. It is described that ~70 nucleotide sugars were identified in bacteria (Decker and Kleczkowski, 2019), and this number could be higher if we consider (*i*) bioprospecting of metagenomic data and discovery of new types and families of enzymes or (*ii*) secondary activities and/or promiscuity of enzymes producing molecules at sub-optimal metabolic concentrations (Peracchi, 2018; Rosenberg and Commichau, 2019; Copley and Copley, 2020). The former requires the study of uncharacterized nucleotide-sugar PPases and the latter to revisit the kinetic properties of already known enzymes. To achieve these tasks, we have reported PPases with new activities (Asención Diez et al., 2010, 2017; Ebrecht et al., 2015; Cereijo et al., 2021), and herein, we deepen the study of promiscuity of the extensively characterized bacterial ADP-Glc PPase (Ballicora et al., 2003).

UDP-Glc and ADP-Glc are synthesized from Glc-1P and their respective nucleoside-triphosphate (NTP; Glc-1P + NTP ↔ NDP-Glc + PPi). In bacteria, the formation of NDP-Glc represents a branching point that determines the fate of Glc in the cell: ADP-Glc is used to form glycogen, while UDP-Glc is shuttled to various metabolic routes (Figueroa et al., 2021). UDP-Glc PPase (EC 2.7.7.9) from prokaryotes has been characterized as a non-regulated enzyme, as is the case for other NDP-sugar PPases. Conversely, ADP-Glc PPase from many bacteria is sensitive to allosteric regulation by intermediates of the organism’s central metabolic pathway(s). Structurally, ADP-Glc PPases are larger proteins than other NDP-sugar PPases, having a C-terminal domain that confers the regulatory function (Ballicora et al., 2003, 2004; Figueroa et al., 2022).

UDP-Glc PPases from different sources were reported to be specific toward the use of UTP and Glc-1P. The only exception so far is the case of a GalU-type enzyme from *Rhodococcus* spp., which exhibits the ability to use GlcN-1P with high efficiency, being the preferred substrate (Cereijo et al., 2021). On the other hand, the ADP-Glc PPase from *E. coli* was found promiscuous toward different NTPs and hexose-1Ps but, when assayed in the presence of allosteric regulators, only Glc-1P and ATP were major substrates (Ebrecht et al., 2017). Thus, the hypothesis regarding the evolution of regulation in ADP-Glc PPases seems to have selected enzymes, which have a preference toward ADP-Glc synthesis after allosteric activation. Interestingly, a divergence had been reported at that time, with the ADP-Glc PPase from *R. jostii* using GlcN-1P as a substrate and its activity responding to Glc-6P, the primary activator of the actinobacterial ADP-Glc PPase (Cereijo et al., 2016). More recently, it was demonstrated that the catalysis of the *R. jostii* enzyme is efficiently enhanced by Glc-6P, Fru-6P, Man-6P, and PEP. The analysis of the regulation of the GlcN-1P allowed the discovery of a new regulating metabolite, GlcN-6P, which exerted activation mainly on the catalysis of the alternative substrate, analogously to the Glc-6P/Glc-1P pair for its canonical activity (Cereijo et al., 2020).

In the work presented here, we extended the analysis of GlcN phosphates regarding their relationships with prokaryotic ADP-Glc PPases either as a substrate (GlcN-1P) or an effector (GlcN-6P). We identified these molecules are related to all the characterized enzymes with common patterns and characteristics depending on the source. Recently, several crystallographic structures were obtained for ADP-Glc PPases (Cupp-Vickery et al., 2008; Hill et al., 2019; Asencion Diez et al., 2020), and they provide invaluable tools to deeper understand the structure–function relationships in the enzyme. We constructed an *in silico* model to understand the structural determinants for substrate specificity related to GlcN-1P, as described below.

The hydroxyl of Glc-1P (or the product ADP-Glc) in position 2 donates a H to the O of the carboxylate from E187 and stands close to a positive density at the N-terminus end of the α-helix F233-H244. This led to the hypothesis that these two major structural elements contribute to selecting a hydroxyl over an amino group, and as such, preferring Glc-1P over GlcN-1P. However, this was not exactly the case. The mutation E187A decreased the overall activity of the enzyme and the apparent affinity for the sugar phosphates, but the selectivity ratio only decreased minimally. This indicates that the H-bond with E187 is a strong feature for the binding of the sugars, but it is not a major contributor in selecting Glc-1P over GlcN-1P. It is well known that at the N-terminus of an α-helix there is an electrostatic positive density that is important for binding of anions (Hol, 1985). This electrostatic interaction most likely contributes to differentially attracting a hydroxyl group over a positively charged primary amine. Experimental evidence that this area attracts negatively charged groups is that in a different subunit of the potato tuber crystal structure, the beta-phosphate of ADP binds there (presented in Figure 3). This α-helix is a common feature of the GT-A fold of the ADP-Glc PPase family (Figueroa et al., 2022), which would explain the widespread selectivity for Glc-1P over GlcN-1P in the family.

Results with the two PPases from *Rhodococci* using GlcN phosphate metabolites led us to propose a putative metabolic node or bifurcation at the level of GlcN-1P, incorporating the characterization of other enzymes, such as GlmU (Cereijo et al., 2021). This enzymological approach led us to revisit GlcN utilization in *Rhodococcus* spp., using *R. jostii* and *R. fascians* as growing models, and to find new metabolic outputs when the microorganisms use GlcN as the sole carbon source (Cereijo et al., manuscript in preparation). Worthy of mentioning, the literature available concerning GlcN utilization by microorganisms is scarce and limited to a group (Flores and Gancedo, 2018), including some Gram-positive bacteria (Gaugué et al., 2013; Uhde et al., 2013; Álvarez-Añorve et al., 2016). Then, given that the allosteric properties of ADP-Glc PPases are linked to the major carbon assimilation pathways(s) in the organisms they belong to, their kinetic and regulatory characterization may help infer metabolic pathways occurring in the organism. In this regard, we extended the study of GlcN metabolites usage by ADP-Glc PPases beyond the *Rhodococci* case.

Our analysis shows that the *K. rhizophila* ADP-Glc PPase follows the general mechanism we previously described for the rhodococcal enzyme (Cereijo et al., 2021), where the activation (either by GlcN-6P or other effectors, e.g., Glc-6P) switches on GlcN-1P consumption. Particularly, the enzyme from *Kocuria* displays this phenomenon in a more accentuated form. The GlcN-6P presence augments activity by two orders of magnitude with a significant increase in the catalytic efficiency toward the alternative substrate. In addition, compared to the protein from *R. fascians*, ADP-Glc PPase from *Kocuria* depicts a higher degree of promiscuity since the selectivity coefficient is 8-fold lower than the former. On the other hand, the *S. coelicolor* ADP-Glc PPase showed a poor use of GlcN-1P, with activity remaining in milliunit values after a weak response to activators. It is possible that these differences could be attributed to the varied degree of sensitivity toward distinct regulatory effectors by the *S. coelicolor* protein compared to the enzymes from *Rhodococci* and *Kocuria* (Asencion Diez et al., 2012; Cereijo et al., 2016, 2020). These particular characteristics in regulation could be related to the many roles proposed for glycogen in Actinobacteria. In *Rhodococci*, the glucan serves as a temporary carbon allocation molecule, closely interrelated to the metabolism of other storage compounds (fatty acids, triacylglycerols) (Hernández et al., 2008, 2019; Hernandez and Alvarez, 2010), whereas in *Streptomyces* spp. glycogen seems to participate (together with trehalose) in processes regarded to cell differentiation (Hey-Ferguson et al., 1973; Brana et al., 1986; Rueda et al., 2001). According to genomic information, *Kocuria* is metabolically similar to *Rhodococci* (Takarada et al., 2008). In this context, the GlcN-1P switch-on mechanism exerted by the corresponding enzyme activators (specifically GlcN-6P) might be a strategy to cope with temporary GlcN phosphates accumulation. It has been demonstrated that increased intracellular hexose-P concentrations could be toxic and detrimental to cell viability (Boulanger et al., 2021).

The hypothetical GlcN-1P utilization “switch-on mechanism” described above is absent in the ADP-Glc PPases other than from *Kocuria* and *Rhodococcus*. Remarkably, little activation was observed in the *R. albus* GlgC and GlgC/GlgD enzymes, although it was already reported they had an inherent low sensitivity to effectors for the canonical substrates (Cereijo et al., 2018). *R. albus* is a microorganism whose ecological niche is the rumen of animals; where GlcN would not be a major carbon source available (Suen et al., 2011). In *A. tumefaciens*, it is well known that the microorganism has Entner-Doudoroff as its central glucose oxidation pathway (Arthur et al., 1973, 1975; Xu et al., 2021); although a transporter system for the GlcN (Zhao and Binns, 2014) and catabolic genes for GlcN utilization was described (Iwamoto et al., 1998).

In *Bacilli*, two different operons related to transport and catabolism of GlcN were reported (Gaugué et al., 2013). The general implication of these pathways is to channel GlcN-6P to Fru-6P, then feeding the core metabolism. The ADP-Glc PPase from *Bacilli* is the only unregulated enzyme of the family, which implies it is “disconnected” from intracellular metabolic signals to modulate glycogen accumulation (Kiel et al., 1994; Takata et al., 1997; Ballicora et al., 2003, 2004). Exploring the role of GlcN-6P as an effector on the active conformations (GlgC and GlgC/GlgD) of *G. stearothermophilus* ADP-Glc PPase reinforced the precedent of the lack of allosteric regulation. However, the features of this enzyme described in this work unmask its capability to utilize GlcN-1P. Remarkably, the modest activity with GlcN-1P shown by the GlgC homotetramer (displaying a clear preference for Glc-1P) is enhanced in the heterotetramer GlgC/GlgD. In this case, the “allosteric behavior” exerted by the GlgD subunit increases the efficiency 60-times for GlcN-1P which is analogous to the “switch-on” mechanism described in the *Kocuria*/*Rhodococcus* enzymes. For the canonical substrate Glc-1P, the *G. stearothermophilus* GlgD subunit increases the catalytic efficiency ~3-fold. Thus, this GlgD behavior resembles the action of the GlgD subunit from *Streptococcus mutans* on the activity with Glc-1P (Asención Diez et al., 2013a). It has already been observed that an ADP-Glc PPase subunit could change the specificity of a ligand. The large subunit of the unicellular algae *Ostreococcus tauri* was considered a “specifier” because its presence in the heterotetramer only increases the specificity for the activator 3-phosphoglycerate (3-PGA) compared to fructose-1,6-bisphosphate (Kuhn et al., 2013). A classic example of a subunit changing substrate specificity was the β-1,4-galactosyltransferase, which changes its specificity from N-acetylglucosamine to glucose when it interacts with the protein α-lactalbumin (Ramakrishnan et al., 2001). The physiological relevance of the increment in GlcN-1P utilization by the GlgC/GlgD enzyme in *G. stearothermophilus* (or other *Bacilli*) remains to be solved; this case also illustrates how enzymological data bring new questions regarding the metabolism while opening the door to novel biotechnological tools.

The ability of the ADP-Glc PPases reported here to utilize GlcN phosphates suggests that evolution could harness the capacity to bind the amino sugar in the substrate pocket even with diminished affinity of the amino group at C2. Also, the allosteric properties of these enzymes can increase catalytic efficiencies. This increased substrate promiscuity could also be considered in terms of underground metabolism. The latter comprises the alternative reactions produced by the promiscuity of some enzymes, which are reactions below the metabolic plausibility (Bar-Even et al., 2011; Davidia et al., 2016; Rosenberg and Commichau, 2019). The underground metabolism serves as an evolutionary tool for the appearance of novel pathways in particular circumstances (D’Ari and Casadesús, 1998; Peracchi, 2018; Rosenberg and Commichau, 2019; Glasner et al., 2020). Works performed in the model organism *E. coli* demonstrated its ability to harness the underground metabolism to discover new metabolic functions (Guzmán et al., 2015). Using this organism, specific examples are in recent works where *(i)* new metabolite links (“serine shunt”) were identified, allowing to rewire central metabolism, particularly the Embden–Meyerhof–Parnas glycolysis (Iacometti et al., 2021) and *(ii)* elucidated the appearance of new pathways for isoleucine biosynthesis (Cotton et al., 2020). *E. coli* was also demonstrated that GlcN-6P intracellular concentration increases two orders of magnitude (up to 9 mM) when the bacterium grows in GlcN as a sole carbon source (Álvarez-Añorve et al., 2009), where specifically a GlcN-6P utilization enzyme [NagB (EC 3.5.99.6)] is activated to channel the hexosamine-P to the central glycolytic pathway (Álvarez-Añorve et al., 2016). However, little is known regarding other metabolites or enzymatic activities linked to GlcN consumption. The properties reported herein for bacterial ADP-Glc PPases would fit this metabolic scenario. Indeed, since there is no enzyme apart from GlmM (EC 5.4.2.10) to GlmU (EC 2.7.7.23/2.3.1.157) reported to use GlcN-1P as a substrate, the allosteric activation of GlcN-1P (either by metabolites or GlgD in *G. stearothermophilus*) may provide the potential for the evolution of alternative/novel GlcN-related pathway(s), to yet unknown intracellular fates. Reinforcing this hypothesis, we recently have published the discovery of a new type of pyrophosphorylase specific for GlcN-1P (Cereijo et al., 2021). Beyond the structural basis for GlcN-P utilization and its putative metabolic implication, we also stress the importance of producing the recombinant enzymes (particularly PPases) to catalyze GlcN-1P to NDP-GlcN since it would be critical for developing different types of molecular tools. As a leading example, we could consider the TDP-Glc PPase from *Salmonella enterica* where is promiscuous activity toward sugar-1P and NTP was deeply studied and exploited to create a library of different NDP-sugars (using natural and non-natural substrates) (Moretti and Thorson, 2007, 2008). The library was proposed as a glycosyl-transferase substrate to modify diverse aglycon molecules (such as antibiotics), by a process-denominated glycorandomization (Křen and Řezanka, 2008). Among the NDP-sugars, a minor group is constituted with amino sugar moieties. Thus, the product of bacterial ADP-Glc PPases catalyzing GlcN-1P may find a niche to be incorporated. The synthesis, purification, and biophysical characterization of these molecules could allow later *in vivo* experiments to establish their potential physiological relevance. In addition, the availability of purified NDP-GlcN may help to identify novel types of (amino)glycosyltransferases (Zhang et al., 2014; Da Costa et al., 2021) and incorporate them into strategies of cell-free glycobiology for the synthesis of novel glycans (Jaroentomeechai et al., 2020).

## Data Availability Statement

The raw data supporting the conclusions of this article will be made available by the authors, without undue reservation.

## Author Contributions

MA, MB, and AI conceived the study, designed the experiments, and analyzed the results. JB, MI, RM, and AC performed the enzymatic characterizations. JB and MB produced the homology model. JB, MA, MB, and AI wrote the manuscript with contributions from all authors. All authors contributed to the article and approved the submitted version.

## Funding

This work was supported by grants from ANPCyT to MA (PICT-2018-00698), to AI (PICT-2017-1515; PICT-2018-00929) and from NSF to MB (MCB 1616851). MA and AI are investigators from CONICET. MI is a Fellow (*Cientibeca*) from UNL.

## Conflict of Interest

The authors declare that the research was conducted in the absence of any commercial or financial relationships that could be construed as a potential conflict of interest.

## Publisher’s Note

All claims expressed in this article are solely those of the authors and do not necessarily represent those of their affiliated organizations, or those of the publisher, the editors and the reviewers. Any product that may be evaluated in this article, or claim that may be made by its manufacturer, is not guaranteed or endorsed by the publisher.

## Supplementary Material

The Supplementary Material for this article can be found online at: https://www.frontiersin.org/articles/10.3389/fmicb.2022.867384/full#supplementary-material
