# Supplementary material for: Carbohydrate Metabolism in Bacteria: Alternative Specificities in ADP-Glucose Pyrophosphorylases Open Novel Metabolic Scenarios and Biotechnological Tools
Source: Front Microbiol. 2022 Apr 27;13:867384. doi: 10.3389/fmicb.2022.867384 (PMC9093745; doi:10.3389/fmicb.2022.867384)
Supplement: Supplementary file 1 [file Data_Sheet_1.PDF]

Supplementary Figure 1: Glc-6P and GlcN-6P effect over the activity of *S. coelicolor* ADP-GlcPPase. Measures were performed with 2 mM ATP and 1.5 mM of the hexose-1P, according to description in Material and Methods.

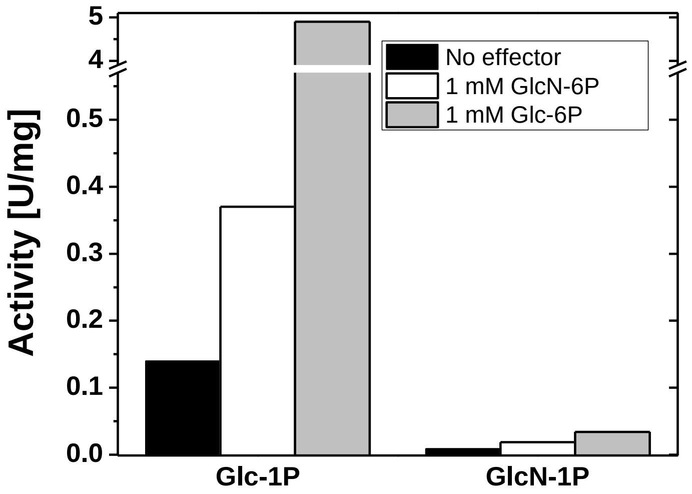

Supplementary Figure 2: ATP curve for the heterotetrameric GlgC/GlgD conformation from *G. stearothermophilus* ADP-Glc PPase. Reaction was performed in presence of 1.5 mM GlcN-1P according to descriptions stated in Material and Methods.

:

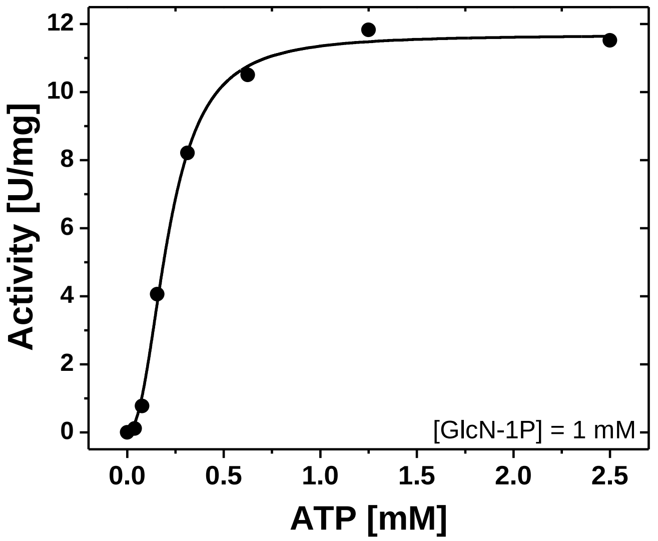

Supplementary Table 1. Comparative Glc-1P and GlcN-1P catalytic efficiencies for bacterial ADP-Glc PPases analyzed in this work. Efficiency was calculated for a single catalytic subunit. Activities were measured in presence of the corresponding activator (depending on the source) and/or in absence of them. ([#]) data adapted from [1]Cereijo et al, 2020, [2] Asencion Diez et al,2012),. n.d., not determined. “Control” refers to absence of any effector.

ADP-Glc PPase source	Effector	Glc-1P efficiency k_{cat}/K_m (s^{-1} mM^{-1})	GlcN-1P efficiency k_{cat}/K_m (s^{-1} mM^{-1})	Ratio efficiencies
R. fascians[#1]	GlcN-6P 2 mM	15.2	0.23	62.08
S. coelicolor	Glc-6P 2 mM	30.59[#1]	n.d	n.d
K. rhizophila	GlcN-6P 2 mM	71.38	8.82	8.09
R. albus GlgC	PEP 10 mM	1.88[#3]	0.03	62.6
R. albus GlgC/GlgD		26.9[#3]	0.43	62.5
G. stearothemophilus GlgC	Control	13.39[#3]	0.36	37.19
G. stearothemophilus GlgC/GlgD		33.05[#3]	22.46	1.47
A. tumefaciens	Control	201.18	1.96	102.64

	Fru-6P<br><br>1.5 mM	1641	21.37	76.79
	Pyr<br><br>1.5 mM	1261	23.76	53.09
